# Achieving ‘coherence’ in routine practice: a qualitative case-based study to describe speech and language therapy interventions with implementation in mind

**DOI:** 10.1186/s43058-021-00159-0

**Published:** 2021-05-26

**Authors:** Avril Nicoll, Margaret Maxwell, Brian Williams

**Affiliations:** 1 Nursing, Midwifery and Allied Health Professions Research Unit, University of Stirling, Stirling, UK; 2 Health Services Research Unit, University of Aberdeen, Aberdeen, UK; 3 School of Health and Social Care, Edinburgh Napier University, Edinburgh, UK

**Keywords:** Coherence, Normalisation Process Theory, Intervention description, Speech and language therapy

## Abstract

**Background:**

Implementation depends on healthcare professionals being able to make sense of a new intervention in relation to their routine practice. Normalisation Process Theory refers to this as coherence work. However, specifying what it takes to achieve coherence is challenging because of variations in new interventions, routine practices and the relationship between them. Frameworks for intervention description may offer a way forward, as they provide broad descriptive categories for comparing complex interventions. To date such frameworks have not been informed by implementation theory, so do not account for the coherence work involved in holding aspects of routine practice constant while doing other aspects differently. Using speech and language therapy as an empirical exemplar, we explored therapists’ experiences of practice change and developed a framework to show how coherence of child speech interventions is achieved.

**Methods:**

We conducted a retrospective case-based qualitative study of how interventions for child speech problems had changed across three NHS speech and language therapy services and private practice in Scotland. A coherence framework was derived through interplay between empirical work with 42 therapists (using in-depth interviews, or self-organised pairs or small focus groups) and Normalisation Process Theory’s construct of coherence.

**Findings:**

Therapists reported a range of practice changes, which had demanded different types of coherence work. Non-traditional interventions had featured for many years in the profession’s research literature but not in clinical practice. Achieving coherence with these interventions was intellectually demanding because they challenged the traditional linguistic assumptions underpinning routine practice. Implementation was also logistically demanding, and therapists felt they had little agency to vary what was locally conventional for their service. In addition, achieving coherence took considerable relational work. Non-traditional interventions were often difficult to explain to children and parents, involved culturally uncomfortable repetitive drills and required therapists to do more tailoring of intervention for individual children.

**Conclusions:**

The intervention coherence framework has practical and theoretical applications. It is designed to help therapists, services and researchers anticipate and address barriers to achieving coherence when implementing non-routine interventions. It also represents a worked example of using theory to make intervention description both user-focused and implementation-friendly.

**Supplementary Information:**

The online version contains supplementary material available at 10.1186/s43058-021-00159-0.

Contributions to the literature

- By focusing on ‘practice change’ rather than implementation of a specific intervention, we have refined our understanding of implementation theory by highlighting the intellectual, logistical and relational work of changing routine practice.
- We have established that it is possible to incorporate this refined implementation theory (via ‘coherence’) in intervention descriptions to draw attention to the key similarities and differences between routine practice and the requirements of new interventions.
- As coherence work went on before, during and after practice change, our study suggests that interpreting coherence as mainly a starting point for implementation may be partly an artefact of research design.

## Background

Recent guidance on intervention development recommends describing the intervention so that people in real-world contexts can change their practice to use it [1]. How to go about describing complex interventions in a way that supports their implementation is less clear.

The Template for Intervention Description and Replication (TIDieR) [2] is the minimum standard for reporting experimental and comparison interventions in trials and systematic reviews. It includes the name of the intervention; why the intervention was used; what materials and procedures were involved; who provided it and how, where, when and how much; any tailoring or modification; and how well (planned and actually) it was delivered.

However, TIDieR may be limited in the support it can provide for practice change as it was designed from an innovator/developer rather than an adopter/implementer perspective [3] without taking account of how interventions, implementers and contexts might interact to make practice change more or less possible [4]. Routine interventions can be entrenched [5] and integrating a non-routine intervention into everyday practice is a social, context-sensitive and dynamic process [3]. Basic technical detail about an intervention is necessary but not sufficient to support the individual and collective work of implementation across a variety of local healthcare contexts where what is ‘routine’ differs [3].

Normalisation Process Theory (NPT) [6] is not a framework for intervention description, but was developed to help explain how healthcare professionals get the work of implementation done. This work includes comparing a new intervention to current practice to make sense of its implications through an individual and collective process of creating ‘coherence’ [6, 7] (Table 1).

**Table 1:** Coherence [6, 7]

‘Coherence’ is one of four core Normalisation Process Theory (NPT) constructs. NPT was developed to highlight that practices (including complex interventions) are ensembles of activity, and that implementing non-routine practices takes individual and collective work. As a middle-range theory (i.e. one designed to guide empirical enquiry in a particular aspect of the social world [41]), NPT is intended to be used flexibly to help explain how people get this implementation work done. NPT’s core constructs cover the different work of implementation: coherence (work to make sense of the job that needs to be done), cognitive participation (the relational work of getting everyone who needs to be involved on board), collective action (working together to make it happen) and reflexive monitoring (working out the value of doing it). The core construct of coherence (sense-making work) has four sub-constructs: • *Differentiation* is about how easily those involved can see that an intervention is different from current ways of working • *Communal specification* is about how well, together, those involved can build a shared understanding of what an intervention is for • *Individual specification* is about how well those who need to be involved grasp what specific tasks and responsibilities they have when using an intervention • *Internalisation* is about how easily those involved notice what value an intervention might bring to their work	

Theorising about how interventions might be described with implementation in mind is particularly important where there is a known gap between interventions shown to be effective and efficient in research contexts and what happens in real-world practice. A good example of this translation gap is speech and language therapy intervention for child speech sound disorders (hereafter referred to as ‘child speech’) [8–10], where efforts to bridge this gap are compounded by high levels of intervention complexity, ambiguity and ambivalence (Additional file 1). This also matters because child speech accounts for almost half of typical community paediatric speech and language therapy caseloads [11].

A mixed method study of speech and language therapy for pre-school children in England involving 245 therapists confirmed that, from their perspective, intervention and its variation is difficult to describe without the caveat of ‘it depends’ [12]. While this suggests therapists put considerable work into creating coherence in everyday practice, we know little about how coherence is achieved when a new intervention is introduced, or how this is affected by context. Therapists’ coherence work may be largely invisible in the sociological sense of informal, behind-the-scenes labour that performs important social functions but may otherwise go unnoticed [13].

### Aim

We sought to make coherence work more visible by identifying what therapists may have to hold constant or do differently to implement non-routine interventions as part of everyday child speech practice in their context. Our research question was: how do speech and language therapists describe the work of integrating complex interventions into their existing practice?

## Methods

We referred to the Standards for Reporting Qualitative Research when drafting this paper [14].

### Research approach

We conducted a retrospective qualitative study of practice change, specifically a case-based sociological inquiry underpinned by the meta-theoretical approach of critical realism [15, 16]. It was retrospective in that the events and outcome (practice change) had already occurred; by offering a ‘best explanation’ for this, we hoped to inform future implementation efforts [17].

**Fig. 1:**
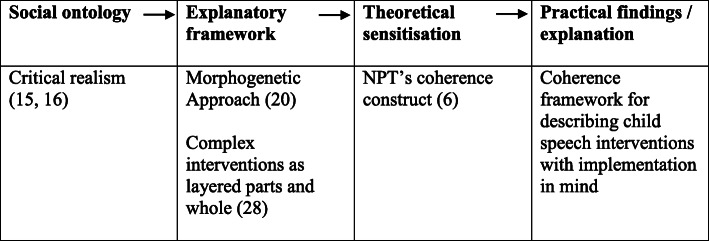
Research approach (adapted from Archer [20])

Through considering the interaction of structure, culture and agency over time, Archer’s Morphogenetic Approach provided a ‘tool kit for developing the analytical histories of emergence’ of practice change ([18], p. 274). We progressed through an interplay between empirical work, based on therapists’ accounts of how they had already changed their practice, and theoretical ideas about implementation, including NPT’s coherence construct. Rather than seeking to test or refine ‘coherence’, we used it to sensitise us to what might be going on empirically that could help us describe child speech interventions with implementation in mind [19]. Figure 1 sets out the research approach, based on a structure proposed by Archer [20].

### Reflexivity

The main author (AN) is a speech and language therapist researcher interested in the work of practice change as a social process. To maintain an implementation-in-practice perspective, she drew on her historical experience as a therapist and editor of a practice magazine and avoided immersion in the child speech intervention research literature. Throughout, she used feedback from co-authors (experienced health service researchers from a sociology background) to reflect critically on how her assumptions may be shaping or constraining the research. This drew her attention to the gap between the research-based idea of a complex intervention as a ‘thing’ to implement and the actual experience of creating coherence in clinical practice.

### Sampling strategy

Sampling was an iterative process of configuring cases (‘casing’) [21, 22]. It involved constantly asking ‘what is this a case of?’ and purposefully seeking data that could reasonably be expected to help test and refine our thinking. Data context was part of the decision-making process. People with actual connections to each other in relation to the research question were sampled to highlight how things ‘got done’ collectively (or did not) and to provide opportunities to corroborate and refine emerging findings [23]. Additional file 2 is a summary of sampling questions and decisions.

The 42 participants came from three NHS speech and language therapy services (anonymised as Blaeshire, Clootshire and Staneshire) and private practice in Scotland. They were sampled ‘in context’ so that actual connections and service structure could also be taken into account [23]. In brief, Blaeshire had invested in a sustained initiative to introduce a range of evidence-based child speech interventions, while a part of Clootshire was exploring ways to deliver greater intensity of intervention for children who needed it. Staneshire and another part of Clootshire were shifting resources away from direct intervention, although therapists could still try out new interventions if they wished. Sampling began chronologically and became more purposive, ending when we judged information power to be adequate [24].

### Technical processes of ethics, data collection and data management

The study was approved by Stirling University’s School of Health Sciences Ethics Committee on 19 November 2014. R&D Management Approval was received from all three participating NHS services by 31 March 2015, and Letters of Access issued.

AN approached speech and language therapy managers of the three services. They agreed to an initial discussion followed by staff meetings to talk about the study and seek permission to contact by email without obligation. All processes were designed to maintain confidentiality, e.g. AN did not discuss with managers or participants who had declined, consented or taken part and offered participants any place, time, or mode of contact.

**Fig. 2:**
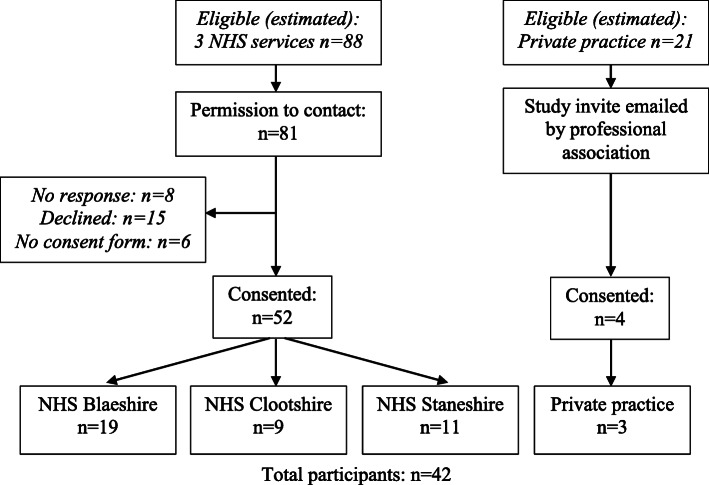
Recruitment flow chart

A recruitment flowchart is shown in Fig. 2. At interview, 9/42 participants were based in Clootshire, 11/42 in Staneshire and 19/42 in Blaeshire and 3/42 were private practitioners. Most elected to have an individual interview (n=28), including one by telephone, while four chose to have paired interviews (n=2) and 10 to participate as small-team focus groups (n=3).

AN conducted all interviews and focus groups. All encounters were audio recorded, with time averaging 78 min (range 48 to 112 min). Some participants provided artefacts, including two protocols developed for parent groups.

Electronic data was held on a secure, password-protected university computer, and paper data stored securely. File labelling did not compromise confidentiality. Digital voice recordings were transferred to the computer as soon as possible, and the recording deleted from the portable device. Electronic data was managed within NVivo 10, Excel and Word.

### Data collection and interplay with theory

AN conducted all contact, interviews and focus groups in a conversational manner. She invited participants to suggest the practice change(s) they wished to discuss and made constant judgements around pausing, probing, reassuring, empathising and encouraging feedback and comparisons.

Interplay with theory was integral to preparing for data collection (see topic guide, Additional file 3) and in how it unfolded. At an early stage of interviewing, AN recognised that NPT’s core construct of ‘coherence’ was central to unpacking the nature of child speech intervention and what it takes to change this in routine practice. Interviews and focus groups were themselves sites of coherence, as participants sought to articulate practice changes and AN encouraged them to consider how a change differed from what they had done before, or how their experience compared to those of others.

### Data analysis

In transcribing all encounters, AN paid attention to accuracy and turn-taking, noting emphasis, hesitation and humour to aid analysis. She used the process of anonymising data to deepen her awareness of how context may have shaped and constrained therapists’ actions. This included giving pseudonyms to participants, services and any non-participants named in interviews; coding the structure of each service; and banding demographic data such as year of qualification and whole-time equivalence.

We used a realist approach to qualitative analysis to make sense of the diversity, distribution and variation in practice change [25]. Rather than developing themes, our analysis focused on describing child speech intervention according to therapists’ reports of how their intervention had—and had not—changed over time in their context.

AN identified, organised and categorised the numerous practice changes raised by therapists in an iterative process. Rather than following the steps of a particular qualitative research method, we compared data and ideas using connecting and categorising activities as responsive ‘moves’ [25]. These included indexes, maps, tables, summaries, ethnographic monologues and a contribution matrix. This led to analytical separation of ‘intervention’ from three other aspects of practice change: the service, caseloads and candidacy. Candidacy [26] refers to who is considered eligible for starting, continuing with and ending therapy. These aspects will be reported in other publications.

We explored the identified ‘intervention’ aspect of practice change further in a number of ways. Two transcripts were intensity sampled for detailed coding because these interviews were nuanced explorations of practice change in relation to complex interventions; the importance of the differentiation component of NPT’s coherence construct was identified in the process of coding the first, with ‘the same yet very different’ a preliminary mechanism. Two documents (group plans) were also compared. Counterfactual thinking [27] took account of absence as well as presence, for example asking ‘What is it about particular interventions that makes them possible (or not) to consider and use?’

AN explored the tension between intervention(s) as parts and a whole [28] through considering interviewees’ experiences of adaptation. These included the concept of fidelity, reasons to adapt, using parts, combining parts of interventions, shifting the weight of routine intervention and de-implementation. She arrived at the coherence framework through progressive ‘casing’ [21].

### Techniques to enhance trustworthiness

AN maintained a critical stance through underpinning questions including ‘What other explanation might there be?’ and ‘Where might I be wrong?’ She recorded developing ideas through memos and sketches, testing them with successive interviewees and two recently retired senior therapists. The co-authors read purposively selected transcripts and regularly offered critical feedback and a different perspective.

Judgement of the adequacy of the sample to help answer the research question informed the decision to stop gathering empirical data [24] (Table 2).

**Table 2:** Judging adequacy of sample by information power [24]

Criterion	Study aim	Sample specificity	Use of theory	Quality of dialogue	Analysis strategy
Lower sample	*Narrow*	***Dense***	***Applied***	***Strong***	*Case*
Higher sample	***Broad***	*Sparse*	*Not applied*	*Weak*	***Cross-case***

## Findings

Participants referred to a pattern of practice, which served as a shared context for how child speech practice as a whole had changed. Sally suggested this pattern was typical around 30 years ago, while Hazel observed laughingly it was ‘what I would have maybe done in 1981?’ Although at that time therapists worked in isolation and had considerable autonomy, intervention had a similar pattern. It focused on one speech sound at a time with a child once a week through a peripatetic service in clinics and schools, with neither parents nor teachers routinely involved.

Change had been driven by a range of factors to which participants had been exposed in a variety of ways. These included developments in policy, the profession and the evidence base; what had happened locally to services over time; practical experience with a range of clients; and education and learning opportunities. Interventions for child speech had become more varied but remained challenging to describe. Aileen was aware of two research initiatives aimed at separating out and specifying interventions but reflected, ‘the more (laughing) I think about it, the more I realise it’s a total mixed bag that I’m using all the time’. Sonia illustrated the fit of the coherence construct when she described eclecticism as not ‘a bit of that and a bit of this’ but a thoughtful combination that ‘amalgamates into the whole really’.

### Coherence work in child speech intervention

Here, we illustrate how context impacted on coherence work when a new intervention was introduced to existing routine practice. Examples of how the four NPT coherence sub-constructs help explain what was happening are highlighted as [*Differentiation*], [*Communal specification*], [*Individual specification*] and [*Internalisation*].

#### Coherence work: non-traditional theory

Participants consistently referred to ‘traditional’ intervention. There was consensus over what it was, and it appeared entrenched. Theories underpinning traditional intervention included attending to how speech sounds are produced (articulation), contrasted (phonology) and manipulated (phonological awareness), as well as to where the speech chain is breaking down (psycholinguistic models).

Non-traditional interventions were a heterogeneous group recognised as ‘new’ to clinical practice within the last 6 years but not to the literature. They challenged traditional linguistic assumptions [*Differentiation*], meaning therapists had to work harder to make sense of and feel comfortable with them. Some reported benefits such as more targeted therapy and faster progress [*Internalisation*].

Therapists who had tried non-traditional interventions expressed surprise and often embarrassment that they had not known about them. On moving to a new service, Erin remembered, ‘It was really eye-opening coming here actually. Cos I just had no…I had no clue and I just thought I can’t believe, you know, that I didn’t know about this’. Wendy returned from maternity leave and noticed ‘a huge, kind of vast change in where everybody’s thinking was now’.

Even where therapists were aware of non-traditional approaches, there was a consistent mismatch with what they saw in practice [*Communal specification*]. This was most evident from more recently qualified participants.

Megan: I remember at university, actually, they talked about doing the complex sounds first? And then that the others would fall into place. It’s one thing I’ve never tried.

Interviewer: And presumably never seen anybody else-

Megan: No… and never really heard anyone else speak about doing it. I just remember it being a suggestion in one of the textbooks.

Traditional elements made theoretical sense to therapists, while non-traditional elements were unsettling [*Individual specification*]. Isla was initially nervous when using an evidence-based intervention where targets are chosen by the child. A 4-year old wanted to say words like ‘waterslide’, ‘karate’ and ‘Cinderella’, “things that you would think from a therapist’s point of view ((puts on fed-up voice)) ‘oh! That’s going to be really hard! She’s not going to manage that’”. Diane repeatedly used the word ‘strange’ to describe a demonstration video of an evidence-based intervention where the therapist did not help the child correct their speech:

Jackie: I find that really hard sitting there

Diane: I thought that was strange

Jackie: Listening to a child.. not achieving for.. for.. (overlapping) a long time

Diane: And you’re saying ‘oh good try’ [[yeah]].. but you’re not really..

Nicole: And if THEY know (laughs), if they know that they’re not achieving, that’s really hard

The entrenchment of traditional intervention in the profession was also confirmed by what happened when it was questioned [*Internalisation*]. Carolyn asked for training in non-traditional approaches, and “it was sort of a bit, as I was told, ‘well that’s your bread and butter’, it’s sort of an assumption that kind of somehow you know everything there is to know”. When Emily was on student placements, “you’re like, ‘so which approach are you using?’, they go ‘oh well I use a combination’ (pause) em, so that was sort of my learning of oh you don’t have to use just one or the other religiously”. Elizabeth interjected phrases such as ‘I feel like I’m a heathen now’ and ‘it’s a bit illegal to say that’ when she wondered whether the tasks that children are traditionally expected to do before they can move on to other ones actually do predict improvement.

The coherence of traditional theories of intervention—and the struggle to find coherence with non-traditional theories—suggests that, as a profession, systematic support is needed for the theoretical work of implementing non-traditional interventions. As discussed in the next section, coherence of logistical work was more dependent on local convention.

#### Coherence work: unconventional logistics

Logistical elements of intervention were where the client was seen (clinic; nursery or school; home), the format (e.g. a group or one-to-one; whether and how parents were involved) and dosage (e.g. number of sessions in a week over what duration). Services introduced unconventional logistics to meet priorities such as reducing waiting lists or shifting resources from specialist to universal provision. However, therapists had little agency to vary where, how and when they saw clients, with implications for coherence of non-traditional interventions.

Therapists often had to experience a change from a conventional to a non-conventional place before they realised the impact it had on other intervention elements [*Internalisation*]. For example, Kate missed the ‘more calming environment’ of a clinic because the busyness, noise and lack of opportunity to involve parents at education premises constrained what intervention was possible. Moving from NHS to private practice gave rise to unexpected benefits when therapists worked in clients’ homes. Isobel noticed ‘I think about the child in the whole, way more than I did before’. She also found it easier to involve parents because “when they are in the situation, you make them think about ‘how can you implement?’”

Where schools had been the conventional place, without parents present, the consequences of a shift to clinics were surprising. Maureen found it helped make the tasks and responsibilities of non-traditional interventions apparent [*Individual specification*]:

With a parent sitting in front of you as well, when you’re asking them to commit to therapy with their child, it almost felt like you wanted to have more of a rationale for what you were doing…

This opportunity to build a shared understanding [*Communal specification*] meant she felt more confident negotiating intensive dosage (‘the evidence for this is this amount of intervention will bring about successes’). Dosage was, however, the logistical convention therapists felt least able to address as shown by observations such as ‘that is how (pause) we’re sort of programmed to be’. Vivienne had experimented with an evidence-based non-traditional intervention, but its required dosage was unconventional for her service [*Differentiation*]:

…it’s meant to have at least 60 minutes a week, and I’m not seeing anybody more than once a week. Most of them are lucky to be seen once a week.

Instead, therapists varied intervention dosage through shortening recommended length, number or frequency of sessions to the local convention and asking parents to do more.

Although participants used parent groups for other client groups, they were unconventional for child speech [*Differentiation*]. Jenna and colleagues now started with two parent group sessions, so parents would see themselves as capable of doing speech work [*Communal specification*]:

…demonstrating all the time how you would carry out these activities with your child. And we have another booklet – we’ve got booklets for everything – that they can go back through and read up on. ‘This is the steps, this is how you do it.’ So we’re trying to be as supportive as possible.

The other child speech parent group also had two sessions but had been introduced to increase throughput, was poorly attended, and therapists saw it as ineffective. Melanie reflected on what was invested in this ‘massive’ service change [*Internalisation*]:

we wanted to try it on a small scale, and maybe try and test that to see if it was effective…but I think there was just high demands from ‘we just need to do this’ and get it rolled out across. So it’s always trying to balance that out and, you know, are we being effective against ‘oh we just need to see these people and get them off the waiting list’

Non-traditional interventions were not used either in a parent group, or when intervention was delegated to parents or education staff. The next section helps explain why they may have been unsuitable for these formats.

#### Coherence work: relating non-traditional interventions to clients

Speech and language therapists manage cases (children and their parents) and caseloads. Non-traditional interventions challenged the coherence of this relational work.

Therapy for child speech necessarily involves meta-language (talking about talking). It became clear that this applied both to talking about the child’s speech problem and talking about a particular intervention in ways that made sense to people who don’t have specialist linguistic knowledge [*Communal specification*]. Louise drew attention to this problem with non-traditional interventions:

How are you gonna pass that on to parents? (laughs) How are you gonna explain? ‘Cos I think some of these concepts… these approaches are very complicated. (pause) REALLY complicated… So it’s ultimately down to the, you know, it’s down to your skills in terms of how you’re able to present that…

Although many participants had spent time learning about non-traditional interventions, the meta-linguistic demands were not addressed in intervention descriptions. How non-traditional interventions impacted on routine session plans also appeared relatively invisible from the literature. Implementing such interventions made Erin ‘realise how set in your ways you get’ and how traditional intervention had ‘this same same same same session plan that you go along with’. She wondered how it was possible that she was still doing similar activities for non-traditional intervention yet ‘every session for every child is totally different?’ [*Differentiation*].

The need to individualise non-traditional interventions also extended to therapy materials [*Individual specification*]. Traditional intervention lent itself to pre-prepared generic sets of materials sorted into folders and boxes for easy transport and adaptable use. As Fran said, ‘I could just grab that’ whereas preparing materials for non-traditional approaches was ‘very very time-consuming’ and ‘there’s no way that you just would grab – d’you know?’ However, it helped that a favourite source of child speech materials (Black Sheep Press) has resources which can support implementation of non-traditional intervention targets. On placing a recent order, Jess was amused when confronted with the previous pattern: ‘you can totally see how your thinking’s changed!’

The clearest relational challenge to coherence of non-traditional interventions came from the impact on support and feedback strategies therapists used to guide the child through therapy. Therapists talked about routinely shifting the power of the relationship to give the child control and being facilitative and non-directive. However, non-traditional interventions often called for directive techniques such as repetitive practice (drilling). Because it was important to make therapy fun and interesting—possibly for themselves as well as clients—therapists were conflicted by the idea of drilling [*Internalisation*]. Beverly acknowledged, ‘it is drills, it is repetitive, it’s not the most exciting therapy work’. Not only was it unexciting in itself, but implementing it reduced the kind of rewards that could be used; as Heather observed, ‘it’s hard because the more exciting you make it the longer it becomes!’ Elizabeth had made a conscious decision to use drilling as part of implementing non-traditional interventions but noted it ‘seems more acceptable to drill in American texts than it is here’, adding:

I don’t know, it’s funny isn’t it? It’s maybe a feeling that you’re doing something to the child and…it’s like making the child into some passive recipient…

Where there is a strong rationale for drilling, this cultural barrier may need to be explicitly addressed.

### Framing coherence in child speech intervention

To make the coherence work described by therapists more visible, we configured an explanatory framework. It is based on what participating therapists had changed or held constant in child speech interventions, taking account of how this was shaped and constrained by context. The framework has three inter-related parts (Table 3):

- Ten changeable elements of child speech intervention
- Binary contextual characteristics that made coherence work more or less challenging
- The main types of work therapists had to do to deliver or change these elements: theoretical (intellectual), logistical (organisational) and relational (people) work

**Table 3:** Child speech intervention coherence framework

Work	Element	Brief definition of element	How context impacts on coherence
Theoretical	Approach	Theory of an intervention’s power to effect change in speech	Traditional/non-traditional
	Target	Sounds child is asked to work with	Traditional/non-traditional
	Focus	Tasks child is asked to do (e.g. listen, compare, produce words)	Traditional/non-traditional
Logistical	Place	Where a child is seen for intervention	Locally conventional/locally unconventional
	Format	How people are involved (e.g. alone or group, parents, assistant)	Conventional/unconventional
	Dosage	The idea that quantity of intervention can make a difference (e.g. how much, how often, how repetitively, for how long)	Conventional/unconventional
Relational	Meta-language	Shared way of talking about speech sounds and intervention	Specified/unspecified
	Scaffold	How behavioural techniques are used to support progress	Congruent/incongruent
	Session	How intervention is structured within each visit	Routine/non-routine
	Material	How things are used to make intervention fun	Adaptable/individual

In summary, theoretical coherence work was increased if elements of intervention were non-traditional for the speech and language therapy profession. Logistical coherence work was increased if elements of intervention were unconventional for the local service. Relational coherence work was increased if therapists had to do more tailoring of intervention elements. Overall, the framework shows that a new intervention demands the most coherence work when it needs a non-traditional approach, target and focus, an unconventional place, format and dosage, and comes with an unspecified meta-language, an incongruent scaffold, a non-routine session plan and the need for individualised materials.

## Discussion

Our intervention coherence framework describes what therapists may have to do differently to implement a non-routine intervention as part of everyday child speech practice in their context. It was generated through a retrospective qualitative study of practice change with child speech therapy as an intensive case [29]. Because it was informed by implementation theory, the concept, approach and aspects of the findings may have transferability to other healthcare professionals who use complex behavioural interventions as well as speech and language therapists who work with other client groups.

Although we studied implementation, our theoretical lens was more social science than implementation science [30]. Social science has provided considerable insights into the complexities of describing clinical practice and accounting for the influence of context. Gabbay and le May’s ethnography with clinicians in primary care [31], for example, identified the ‘mindlines’ that make practice happen and provide a vehicle for changing it. Cristancho et al. [32], in recognising that procedural aspects of surgery are easier to describe than the human experience, used ‘rich pictures’ to reveal the social, cultural and personal influences at play.

Reconciling interventions as ‘things’ which can be described with how implementation is experienced in routine practice is, however, an ongoing methodological challenge. Intervention components have more or less plasticity to be moulded, contexts more or less elasticity to accommodate new interventions, and healthcare professionals have to maintain a service while implementing non-routine interventions [33]. Instead of beginning with a description of evidence-based complex interventions and examining how they were implemented (or not), we therefore began by exploring what practice changes had already been possible (or not), and how this varied by context (or did not).

Importantly, our sampling strategy ensured that ‘what had already been possible’ included non-traditional interventions which had been identified by the majority of respondents to a recent UK survey as rarely/never used in practice [9]. Innovator-focused intervention description gives priority to technical detail about ‘how it ought to be done’, while adopter-focused description is about ‘what it might take to get it done’. In shifting the empirical focus of description efforts from the innovator (developer) to the intended adopter (user), we are also responding to Horton et al.’s [3] argument for research approaches that might increase the likelihood of intervention spread.

Coherence is one of four dynamically interacting NPT mechanisms underpinning implementation, along with cognitive participation, collective action and reflexive monitoring. A systematic review of how NPT has been used in feasibility studies and process evaluations of complex healthcare interventions (n=108) identified that, of the four, coherence was mainly seen as ‘an obligatory point of departure for implementation processes…sometimes at the expense of other activities’ ([34], p. 15). We selected coherence for its relevance to describing interventions with implementation in mind. This does not imply that it preceded or was more important than the other NPT mechanisms for practice change; indeed, much sense-making was only possible for participants through the doing. It may be that the characterisation of coherence as mainly a ‘pivotal first stage’ ([35], p. 104), ‘a planning phase’ ([36], p. 220) and ‘a starting point’ ([37], p. 4/14) is partly an artefact of implementation study designs, i.e. because they track implementation of a ‘thing’, rather than how coherence is achieved in routine practice where a variety of ‘things’ may or may not have been introduced.

An NPT-informed retrospective qualitative study of implementation of a shared decision-making programme identified that coherence was often missing [38]. As implementation is more complex than delivering an intervention to patients, the authors concluded “How to achieve ‘coherence’ in practice is the next logical research question” ([38], p. 7). Linking the coherence construct with intervention description from an implementer perspective is a step in that direction.

As with all frameworks, the number and scope of elements is a fallible and somewhat arbitrary effort to balance level of nuance with purpose, i.e. differences that matter in practice. The intervention coherence framework has 10 elements, with contextual considerations presented as binaries. By indicating the main type of work required, it makes explicit that implementation depends on addressing routines and assumptions of the profession and services, not just therapists. As well as supporting intervention developers to have implementation in mind [1], it could be used by services to map the extent and type of work and support a particular intervention would demand. Based on our participants’ experience, this could include specifying elements of coherence where barriers might not have been anticipated (e.g. session planning or incongruent therapeutic behaviours). In addition, our inclusion of the meta-language element chimes with Morgan et al.’s finding that parent understanding of intervention is important to therapists but under-investigated in the research literature [12].

Our adopter-focused coherence framework is complementary to innovator-focused intervention description and it is instructive to consider whether these different perspectives make a difference to the content. The intervention coherence framework explicates the ‘what’ and ‘tailoring’ elements of intervention in the generic TIDieR framework [2] but omits ‘how well’. With 72 elements, the child speech-specific Phonological Intervention Taxonomy [39] is more detailed and, like TIDieR, is focused on following procedures with fidelity. However, its 15 subcategories highlight the importance of linguistic knowledge, logistical considerations, people skills and materials, all of which are covered in the intervention coherence framework.

The novel and transferable contribution of the intervention coherence framework is less in its identified elements of intervention than the way it relates these to the dynamic work and context of implementation. This may be particularly helpful where there is intervention complexity, ambiguity and ambivalence. A recent scoping study, for example, used qualitative methods to make sense of the approaches, practices, service models and ways of working that characterise physiotherapy, occupational therapy and speech and language therapy for children with neuro-disability [40]. Conclusions emphasised the state of dynamic change and development the professions are operating in, the heterogeneity of the client group, the many potential ‘active ingredients’ and uncertainty over mechanisms of change.

This brings us to a final observation. Understandably, definitions of the ‘differentiation’ component of NPT’s coherence construct emphasise the importance of specifying differences or uniqueness in a new intervention compared to current practice [36]. However, our findings suggest that, given the ongoing challenge of specifying and achieving coherence in practice, a descriptive framework which identifies what could be held constant is as important as identifying what needs to change.

### Limitations

A key strength of this study (sampling) is also a limitation, as we purposively sought the perspective of therapists and have therefore not accounted for the coherence work this creates for other people such as assistants, educators, parents or children. To ensure actual connections, sampling was limited to Scotland, so it is unclear to what extent these particular practice changes apply to practice change elsewhere. In addition, to achieve a practice perspective, it was important to move outside the frame of evidence-based practice, so our decisions to include practice changes were not dependent on their proven effectiveness. This is however mitigated by the inclusion of Blaeshire, where there had been a planned programme to implement non-traditional evidence-based interventions.

## Conclusion and recommendations

So, how can we describe complex interventions in a way that supports their implementation?

Our findings confirm that coherence work is both integral to clinical practice and central to changing it. They take into account that healthcare professionals, professions and services have different starting points for practice change. They also extend practical application of NPT’s coherence construct to the field of intervention description.

The intervention coherence framework comprises what our research suggests should be discussed if individual and collective coherence is to be achieved when implementing non-routine child speech interventions. Our intention was to encourage new ways of thinking about how interventions can be described with implementation in mind. However, a number of participants referred to making or finding simple plotting devices helpful for decision making. We have therefore laid the framework out for testing as a heuristic tool (Additional file 4) to aid decision making and planning around implementation.

## Supplementary Information

**Additional file 1.** Child speech as an information-rich field.

**Additional file 2.** Sampling questions and decisions.

**Additional file 3.** Topic guide.

**Additional file 4.** Mapping challenges to child speech intervention coherence.

## Data Availability

The signed participant consent form included item 6: ‘I understand that at the end of the study anonymised transcripts will be donated to a secure archive for future use by researchers.’ This will be done when publications are complete. This paper builds on a thesis where further data is available: Nicoll A. Speech and language therapy in practice: a critical realist account of how and why speech and language therapists in community settings in Scotland have changed their intervention for children with speech sound disorders. http://hdl.handle.net/1893/27257: University of Stirling; 2017.

## Abbreviations

NPT: Normalisation Process Theory
TIDieR: Template for Intervention Description and Replication
