# Supplementary material for: Achieving ‘coherence’ in routine practice: a qualitative case-based study to describe speech and language therapy interventions with implementation in mind
Source: Implement Sci Commun. 2021 May 26;2:56. doi: 10.1186/s43058-021-00159-0 (PMC8157687; doi:10.1186/s43058-021-00159-0)
Supplement: Supplementary file 1 — Additional file 1. Child speech as an information-rich field. [file 43058_2021_159_MOESM1_ESM.docx]

# Additional file 1 Child speech as an information-rich field

NPT’s notion of coherence suggests that an intervention description framework may offer greater support for implementation if it is structured according to *what professionals might have to do differently in their context and setting*. We developed this idea while exploring implementation in speech and language therapy for child speech. Speech and language therapists intervene to improve the lives of people of all ages who have difficulties with communication or swallowing. They are the fourth largest group of allied health professionals (1). Children with speech problems are a high usage client group; in a UK study, they made up almost half the typical caseload of community generalist paediatric therapists (2).

Applying the principle of intensity sampling (3), child speech was an information-rich field to develop an intervention coherence framework as it is characterised by intervention complexity, intervention ambiguity, and intervention ambivalence.

### Intervention complexity

Child speech interventions are at the high end of the intervention complexity spectrum. They have multiple components, depend on client participation, professional judgement and human interaction over time, and have important but ambiguous outcomes (4). Theorised by Clark (5), a healthcare intervention may have a simple component or a complicated sequential structure and lead to a predictable outcome. More usually, as in child speech, intervention complexity produces a range of intended and unintended consequences for different people in different circumstances.

Such complex interventions have many component parts which interact in more and less important ways and come together as a whole (5). It may not be obvious which components and interactions are necessary or sufficient for a complex intervention to have its intended effect when implemented, or what can be added, adapted or removed to improve (and not reduce) its effectiveness in clinical practice.

An indication of complexity comes from the number of elements identified by a group of expert child speech researchers. They conducted a content analysis of 15 empirically supported interventions to develop a descriptive intervention taxonomy (6). This included 72 intervention elements, with the range of required or optional elements per intervention ranging from 27 to 59.

### Intervention ambiguity

In the child speech field alone, intervention ambiguity means speech and language therapists have “a smorgasbord of approaches to choose from” (7), p.156. Baker and McLeod (8) conducted a comprehensive narrative review that identified 46 distinct child speech intervention approaches from 135 studies (1979 to 2009). They concluded that intervention is more effective than none and that therapists lack comparative research to guide choices. The authors of a forthcoming Cochrane review of child speech and language interventions opted to include only trials of an active intervention versus control. They argued this was necessary for interpretation and coherent reporting, given the “array of different alternative interventions” (9), p.8/41.

In both research and practice, ambiguity of evidence is compounded by sub-optimal intervention description. Relatively few child therapy interventions are well-specified through a manual (10). A literature review and empirical work for the Child Talk study confirmed that intervention description lacks detail and consistency and that making this tacit knowledge explicit is an ongoing challenge (11), (12). This work built on a previous finding that, in practice settings, therapists tended to describe their intervention in terms of activities and resources (13).

### Intervention ambivalence

Finally, although the notion of complex interventions has gained traction in healthcare research, therapists may be ambivalent about its relevance for their practice. A rare sociological study of the work of speech and language therapists in the UK (14) drew on Foucault’s ideas, practice guidelines, and 33 interviews. Interviewees stressed the uniqueness of both their role and of clients, meaning that the “work involved was different for every client, family and circumstance” (p.5). This importance that therapists report placing on individualisation of intervention was a key finding from the Child Talk exploratory mixed method research with 245 therapists in England (11). Studying speech and language therapists in Ireland’s reasoning for treatment choices through three focus groups (48 participants), McCurtin and Carter (15) also found practice was “pivoted” on individual clients. These therapists used a toolkit analogy and discussed having an array of ingredients to combine eclectically rather than recipes to follow. In addition, surveys of therapists about their child speech practices (e.g. (16) (UK), (17) (US), (18) (AUS)) paint a picture of eclectic practice shaped by cultural and service context, and of intervention practices limited by constraints on service delivery.

# References

1. NES. Allied health professions education and workforce report: speech and language therapy. <https://www.nes.scot.nhs.uk/media/2491052/slt_final_2013oct.pdf> (Accessed 17 May 2020): NHS Education for Scotland; 2013.

2. Broomfield J, Dodd B. Children with speech and language disability: caseload characteristics. International Journal of Language & Communication Disorders. 2004;39(3):303-24.

3. Patton MQ. Purposeful sampling. Qualitative evaluation and research methods. Beverly Hills, CA: SAGE; 1990. p. 169-86.

4. Wells M, Williams B, Treweek S, Coyle J, Taylor J. Intervention description is not enough: evidence from an in-depth multiple case study on the untold role and impact of context in randomised controlled trials of seven complex interventions. Trials. 2012;13(1):95.

5. Clark AM. What are the components of complex interventions in healthcare? Theorizing approaches to parts, powers and the whole intervention. Social Science & Medicine. 2013;93:185-93.

6. Baker E, Williams AL, McLeod S, McCauley R. Elements of phonological interventions for children with speech sound disorders: the development of a taxonomy. American Journal of Speech-Language Pathology. 2018;27(3):906-35.

7. Baker E. Management of speech impairment in children: the journey so far and the road ahead. Advances in Speech Language Pathology. 2006;8(3):156-63.

8. Baker E, McLeod S. Evidence-based practice for children with speech sound disorders: part 1 narrative review. Language, Speech, and Hearing Services in Schools. 2011;42(2):102-39.

9. Law J, Dennis JA, Charlton JJV. Speech and language therapy interventions for children with primary speech and/or language disorders. Cochrane Database of Systematic Reviews. 2017(1).

10. Beresford B, Clarke S, Maddison J. Therapy interventions for children with neurodisabilities: a qualitative scoping study. Health Technology Assessment. 2018;22(3).

11. Morgan L, Marshall J, Harding S, Powell G, Wren Y, Coad J, et al. ‘It depends’: Characterizing speech and language therapy for preschool children with developmental speech and language disorders. International Journal of Language & Communication Disorders. 2019;54(6):954-70.

12. Roulstone SE, Marshall JE, Powell GG, Goldbart J, Wren YE, Coad J, et al. Evidence-based intervention for preschool children with primary speech and language impairments: Child Talk - an exploratory mixed-methods study. Programme Grants Appl Res. 2015;3(5).

13. Roulstone S, Wren Y, Bakopoulou I, Lindsay G. Interventions for children with speech, language and communication needs: An exploration of current practice. Child Language Teaching and Therapy. 2012;28(3):325-41.

14. Butler C. Working the 'wise’ in speech and language therapy: evidence-based practice, biopolitics and ‘pastoral labour’. Social Science & Medicine. 2019;230:1-8.

15. McCurtin A, Carter B. ‘We don't have recipes; we just have loads of ingredients’: explanations of evidence and clinical decision making by speech and language therapists. Journal of Evaluation in Clinical Practice. 2015;21(6):1142-50.

16. Joffe V, Pring T. Children with phonological problems: a survey of clinical practice. International Journal of Language & Communication Disorders. 2008;43(2):154-64.

17. Brumbaugh KM, Smit AB. Treating children ages 3-6 who have speech sound disorder: a survey. Language, Speech, and Hearing Services in Schools. 2013;44(3):306-19.

18. McLeod S, Baker E. Speech-language pathologists’ practices regarding assessment, analysis, target selection, intervention, and service delivery for children with speech sound disorders. Clinical Linguistics & Phonetics. 2014;28(7-8):508-31.
