# Supplementary material for: Achieving ‘coherence’ in routine practice: a qualitative case-based study to describe speech and language therapy interventions with implementation in mind
Source: Implement Sci Commun. 2021 May 26;2:56. doi: 10.1186/s43058-021-00159-0 (PMC8157687; doi:10.1186/s43058-021-00159-0)
Supplement: Supplementary file 2 — Additional file 2. Sampling questions and decisions. [file 43058_2021_159_MOESM2_ESM.docx]

# Additional file 2 Sampling questions and decisions

| **Sampling questions** | **Sampling decisions** |
| --- | --- |
| *Who has most in-depth experience of child speech practice (and therefore potentially of practice change)?* | Community speech and language therapists |
| *Where will I find these people, with actual connections and potential diversity of relevant experience built in?* | NHS areas in Scotland (where there is anecdotal reason to think there will be different experiences of practice change), and private practice |
| *What social circumstances are likely to have contributed to diversity of practice change experience?* | Whole time equivalent; NHS banding; qualification place and level; age; career pattern; caseload type; job role; student supervision; training in child speech |
| *How can I structure data collection to make the most of actual social connections?* | Offer options of individual interviews or self-generated pairs or focus groups |
| *What framing is most likely to generate an implementation-in-practice perspective?* | Experiences of ‘practice change’ (not of ‘evidence-based practice’ or of implementing specific named interventions) |
