# Supplementary material for: Achieving ‘coherence’ in routine practice: a qualitative case-based study to describe speech and language therapy interventions with implementation in mind
Source: Implement Sci Commun. 2021 May 26;2:56. doi: 10.1186/s43058-021-00159-0 (PMC8157687; doi:10.1186/s43058-021-00159-0)
Supplement: Supplementary file 3 — Additional file 3. Topic guide. [file 43058_2021_159_MOESM3_ESM.docx]

**Topic Guide**


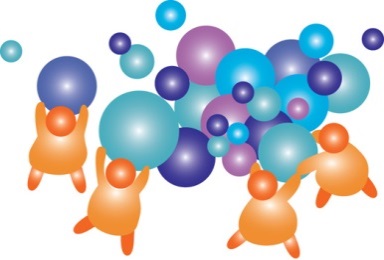


**A qualitative research study with community**

**speech and language therapists:**

**‘What does it take to change your practice?’**

| **Topics** | **Exemplar questions** |
| --- | --- |
| - Priorities | 1. What practice changes would you like to tell me about? 2. What was your role (job) at the time of each? 3. Which is most important to you? / Which would you like to start with? |
| - Social context - Ideas about practice - Ideas about agency (individual / collective) - Time | 1. What were you already doing (to deal with this kind of problem)? 2. Who (or where) did the idea for the change come from? 3. How was it different from what you were already doing? / What did you have to do differently? 4. What happened to this idea? What did you do with it? How did you feel about it? What were other people (who?) saying? How did that affect your thinking? 5. What would be happening now if this idea had never reached you? |
| - Reasons for action (individual / collective) - Ideas about practice, processes, resources - Ideas about power - Time | 1. Why did you act on this idea (at this time)? 2. What did you have to do to put it into practice? / How did you manage to get it to happen? How did you feel about it at the time? 3. Who (or what) helped you, and how? 4. What reactions did you get (and from whom)? How did that affect your thinking? 5. What ‘things’ had to change too (assessments, materials) and what / who did that involve? 6. How long did this take? 7. What adaptations did you make? Why? How? |
| - Reasons for action (individual / collective) - Ideas about practice, roles - Social context | 1. Why did you stick with it (the change)? How did you know it was going to stick? 2. If you had been in a different role at the time, could it still have happened? Why / why not? 3. Could you have gone further (with the change)? Would you go back now? 4. What other consequences, if any, has this change had for your practice? For your clients? 5. Who else knows about this change? How do they know? When did they know? 6. Why does this change stand out in your mind? |

Note: The topic areas are drawn from the conceptual framework for this study, which is based on de Souza’s (2013) interpretation of Margaret Archer’s Morphogenetic Approach (1995).

**References**

Archer, M.S. (1995) *Realist social theory: the morphogenetic approach.* Cambridge: Cambridge University Press.

de Souza, D.E. (2013) Elaborating the Context-Mechanism-Outcome configuration (CMOc) in realist evaluation: A critical realist perspective. *Evaluation,* 19 (2), pp. 141-154.
