# Supplementary material for: Achieving ‘coherence’ in routine practice: a qualitative case-based study to describe speech and language therapy interventions with implementation in mind
Source: Implement Sci Commun. 2021 May 26;2:56. doi: 10.1186/s43058-021-00159-0 (PMC8157687; doi:10.1186/s43058-021-00159-0)
Supplement: Supplementary file 4 — Additional file 4. Mapping challenges to child speech intervention coherence. [file 43058_2021_159_MOESM4_ESM.docx]

##### Additional file 4 Mapping challenges to child speech intervention coherence

| Element | Current practice | | Mapping coherence of a new intervention | |
| --- | --- | --- | --- | --- |
| **Approach** | Traditional | Non-traditional | Traditional | Non-traditional |
| **Target** | Traditional | Non-traditional | Traditional | Non-traditional |
| **Focus** | Traditional | Non-traditional | Traditional | Non-traditional |
| **Place** | Locally conventional | (Locally unconventional)* | Locally conventional | Locally unconventional |
| **Format** | Conventional | Unconventional | Conventional | Unconventional |
| **Dosage** | Conventional | Unconventional | Conventional | Unconventional |
| **Meta-language** | Specified | Unspecified | Specified | Unspecified |
| **Scaffold** | Congruent | (Incongruent)* | Congruent | Incongruent |
| **Session** | Routine | (Non-routine)* | Routine | Non-routine |
| **Material** | Adaptable | Individual | Adaptable | Individual |

**Unlikely to be applicable to current practice*
